# Understanding Bystander’s Response and Intentions in Non-Consensual Sexting: Insights from the Theory of Normative Social Behavior

**DOI:** 10.1007/s10508-025-03384-z

**Published:** 2026-03-24

**Authors:** Daniela Villa-Henao, Mónica Ojeda, Noelia Muñoz-Fernández, Rosario Del Rey, Joaquín A. Mora-Merchán

**Affiliations:** Department of Developmental and Educational Psychology, Universidad de Sevilla, C/Camilo José Cela, s/n, 41018 Seville, Spain

**Keywords:** Non-consensual sexting, Theory of normative social behavior, Bystander response, Social norms, Empathy, Sexting

## Abstract

Non-consensual sexting entails the dissemination of erotic-sexual content without the consent of the original sender. Previous research has mainly focused on understanding the behavior of victims and aggressors. However, bystanders have been found to exert a key role in the psychosocial dynamics of this type of cyberviolence. Thus, it is essential to enhance our understanding of bystander response patterns by examining theoretical frameworks conducive to their explanation. This study contributes to this endeavor by assessing the applicability of the theory of normative social behavior (TNSB) in understanding bystanders’ responses and intentions within the context of non-consensual sexting. Data were collected from 2539 students (49.2% girls, 49.1% boys, 1.7% others) aged 11–18 years. Employing structural equation modeling, the results of this study suggest that the social norms that constitute the TNSB framework provide a solid basis for understanding bystanders’ responses and intentions to non-consensual sexting. Specifically, friends’ injunctive norms, subjective norms, and descriptive norms were key predictors of bystander responses. Incorporating additional factors, such as empathy, heteronormative attitudes and beliefs, and parental supervision significantly improved model fit. Empathy was positively related to defensive responses and negatively to passivity. Heteronormative attitudes and beliefs were significant predictors across all three response types. Parental supervision was positively associated with defensive responses and negatively with passive ones. These findings offer a comprehensive framework for understanding adolescent bystander behavior in non-consensual sexting scenarios and underscore the importance of targeting both social and individual factors in prevention and intervention efforts.

## Introduction

Sexting, defined as the exchange—i.e., the sending, receiving, or forwarding—of erotic sexual content over the Internet (Klettke et al., 2014; Strassberg et al., 2013), allows adolescents and young people to explore their sexuality in the virtual sphere (Mosley & Lancaster, 2019). However, when this exchange takes place without the permission of the original sender (i.e., non-consensual sexting [NCS]), it constitutes a form of sexual violence or abuse on its own (Walker & Sleath, 2017). Although NCS involves the dissemination of erotic-sexual messages, images, or videos, it is important to note that it is different from pornography. The latter is a broader term that refers to the distribution of sexually explicit material through various media, such as films, stories, or images, made explicitly for pleasure and which may or may not involve consent (Jacobs, 2004). In contrast, NCS specifically involves the unauthorized sharing of personal erotic-sexual content, referring to cases in which erotic-sexual material is forwarded without the consent of the person depicted through the internet, constituting an act of harassment and/or violence (Barroso et al., 2023; Ganson et al., 2024; Mishna et al., 2023; Morelli et al., 2016; Ringrose et al., 2012).

According to a recent meta-analysis by Mori et al. (2022), the prevalence of non-consensual forwarding is as high as 14.5%, which reflects the normalization of this behavior among adolescents. This phenomenon carries significant psychological, social, and legal consequences for the people involved (Judge, 2012; Wachs et al., 2021). Moreover, NCS have been positively related to depressive symptoms, non-suicidal self-harm, and other sexual risk behaviors (Wright & Wachs, 2024); highlighting the pressing need to investigate the underlying factors driving involvement in such acts. Contemporary research has predominantly focused on understanding NCS from the perspectives of victims and aggressors (Barrense-Dias et al., 2020; Hollá et al., 2018; Van Ouytsel et al., 2017b). For instance, excessive use of the internet and social media, along with being female and younger in age, have been identified as significant predictors of victimization related to risky sexual behaviors (Pistoni et al., 2023; Tamarit et al., 2021). On the other hand, motivations such as revenge, fun, the need for popularity, and even sextortion are frequently cited as drivers of non-consensual forwarding of explicit content among young people (Barrense-Dias et al., 2020; Naezer & Van Oosterhout, 2021; Patchin & Hinduja, 2020).

Similarly, efforts in the field of online sexual violence prevention have often focused on the roles of victims and perpetrators, with the primary goal of mitigating associated risk factors. Although well-intentioned, these approaches often oversimplify the complexities of these phenomena, reinforce gender norms, and neglect broader social dynamics, frequently falling into victim-blaming discourses (Krieger, 2017; Naezer & Van Oosterhout, 2021). These perspectives often perpetuate strategies that judge the sexuality of adolescents, attributing to them undue responsibility in the dissemination of their own content (Krebbekx, 2024). Rather than blaming victims for their engagement in sexual behavior, research, and prevention efforts should include broader perspectives that transform traditional approaches to NCS prevention and include other relevant actors, such as bystanders (Maes et al., 2024).

### Bystanders’ Intervention Toward Non-consensual Sexting

Bystanders may play a crucial role in the widespread forwarding of intimate images, as their actions—such as encouraging or validating the aggressors—may contribute to perpetuating this harmful behavior. Understanding bystanders’ behavior and their intentions to respond is therefore essential for designing effective interventions to prevent the non-consensual forwarding of such content. Studies such as Salmivalli et al. (1998) revealed that peers acting as bystanders to violent behaviors can perform diverse actions ranging from defending the victim to passivity or inaction, or even to reinforcing the aggressor. In the specific context of NCS and related forms of online sexual violence, bystanders’ actions and intentions could also fit into the previous categories (Barrense-Dias et al., 2024; Mainwaring et al., 2023b). Defensive responses toward NCS may include actions such as reporting the incident to an authority, confronting the aggressor, or providing support to the victim. In contrast, reinforcing responses would include actions that exacerbate the harm inflicted by the aggressor, such as validating the aggressor’s actions by taunting, gossiping, shunning, or insulting the victim. Finally, some bystanders may exhibit passive responses to NCS, characterized by inaction, such as ignoring or avoiding the situation.

Previous research has drawn attention to the importance of bystanders’ intervention in forms of violence analogous to NCS, such as cyberbullying, cyberhate, or image-based sexual abuse (Costello et al., 2023; Mainwaring et al., 2023b; Polanco-Levicán & Salvo-Garrido, 2021; Rudnicki et al., 2023; Sarmiento et al., 2019), highlighting their leading role in the maintenance or cessation of the phenomenon. In particular, bystanders who actively intervene in defense of victims could be a crucial protective factor for those experiencing violence or cyberviolence (Maes et al., 2024). This makes bystanders a critical focus for prevention efforts, as empowering them to intervene effectively may avoid the normalization of these harmful dynamics.

Unfortunately, recent research suggests that bystanders of online sexual violence rarely intervene (Harder, 2021). One possible explanation for this phenomenon is the bystander effect, where individuals are less likely to act in an emergency or problematic situation when others are present (Darley & Latane, 1968). This effect is often driven by a diffusion of responsibility, where people assume that someone else will act, reducing the urgency or perceived necessity of their own intervention. Given the nature of NCS and the unique characteristics of the digital platforms on which they occur, the bystander effect can become especially pronounced, as the virtual environment fosters a sense of anonymity and detachment, often exacerbating inaction (Suler, 2004; Zhong et al., 2020).

In this sense, it is essential to understand the barriers and facilitators that influence bystander intervention in cyberviolence in general, and NCS in particular. Advances in understanding bystanders’ behavior and intentions in response to sexual violence, both online and offline, suggest that the lack of recognition of the problem, the absence of a sense of responsibility, fear of becoming victims themselves, or fear of retaliation from peer groups, are key obstacles to intervention (Barrense-Dias et al., 2024; Burn, 2009). On the other hand, greater empathy toward victims, a higher sense of self-efficacy, and the presence of social norms that support intervention (i.e., when there is a belief that peers endorse such behavior) facilitate positive intervention (Mainwaring et al., 2023a).

Although these findings are valuable for understanding the behavior of bystanders or even their intentions to respond to cyberviolence, and in general provide a useful starting point for understanding bystanders’ intervention, they may be insufficient to explain the unique dynamics that emerge in NCS, the literature on which is still limited. Therefore, the specific study of bystanders in the context of NCS is crucial for contemporary research.

### The Theory of Normative Social Behavior

The application of theoretical models that consider the complexities of NCS may offer an effective approach to shed light on the lack of understanding of the role of NCS bystanders. Previous studies have used theoretical frameworks to predict the involvement of adolescents and young adults in sexting, mainly centered on the theory of planned behavior or social learning theory, highlighting the effectiveness of social norm-based theories in explaining risky behaviors (Dodaj & Sesar, 2024; Dodaj et al., 2023; Walrave et al., 2014). Moreover, social norms have also been used in previous studies to explain phenomena such as unwanted sexual solicitation or sexting, demonstrating that online sexual risk behaviors are positively associated with perceived peer approval (Baumgartner et al., 2011; Lippman & Campbell, 2014; Van Ouytsel et al., 2017a; Wilson et al., 2021). Although these studies represent a significant advancement in understanding the role of social norms in adolescents’ behavior regarding online sexual risks, they may not be specific enough to assert that similar patterns of social norms influence bystander behavior in the context of NCS.

In that context, studying the applicability of the theory of normative social behavior (TNSB) (Rimal & Real, 2005; Rimal & Yilma, 2022), could be a good starting point to address this issue. The TNSB is a psychological approach to explain deviant human behaviors such as alcoholism, sexual aggression, or even unhealthy food consumption (Carcioppolo & Jensen, 2012; Mabry & Turner, 2016; Reynolds-Tylus et al., 2019; Rimal, 2008; Varava, 2019). Therefore, it could be deployed as a suitable framework for analyzing bystander behavioral responses in the context of NCS as a normalized form of cyberviolence (Brighi et al., 2023; Durán & Rodríguez-Domínguez, 2020).

According to the TNSB, interactions among social norms (descriptive, injunctive, subjective, and collective norms) influence individuals’ decision-making and behavior (Rimal & Real, 2005). Descriptive norms refer to people’s perceptions about the prevalence of a behavior in their social sphere (i.e., a description of what is considered normal) (Chung & Rimal, 2016). Several studies have demonstrated that frequent exposure to aggressive behavior can lead individuals to interpret such behavior as typical, which in turn may result in a lack of intervention (Harlow et al., 2021; McMahon & Banyard, 2012). Therefore, it could be argued that the frequency of involvement in NCS provides information about previous experiences and the normalization of the behavior, impacting bystander decision-making.

In contrast, injunctive norms refer to perceptions of the degree of approval or disapproval of a certain behavior by referents or relevant group members, and subjective norms refer to perceptions about what others expect one to do about a particular behavior (Hayashi & Tahmasbi, 2022). In the context of bystanders of NCS, these norms may be inherently linked to broader norms related to online behaviors. Bystanders’ responses to incidents of NCS may depend on how they perceive the attitudes of those in their social environment not only toward the specific act itself but also toward risky behaviors in the digital realm overall (Bastiaensens et al., 2016). Thus, understanding injunctive norms and subjective norms regarding online risky behavior is crucial to comprehend how adolescents navigate the ethical dilemmas arising from the non-consensual forwarding of intimate images online.

Collective norms, on the other hand, are described as “codes of conduct that prescribe or proscribe behaviors that members of a group can implement” (Lapinski & Rimal, 2005, p. 129). The main difference between collective norms and injunctive norms lies in how they are conceptualized and measured (Rimal & Lapinski, 2015). While collective norms operate at the level of the social group or social domain (i.e., representing the code of conduct of a group), injunctive norms operate at the individual level (i.e., representing an individual’s interpretation of the prevailing collective norms) (Lapinski & Rimal, 2005). Therefore, the measurement of collective norms should aim to capture the group dynamics surrounding NCS involvement within the immediate context of the individuals, such as their school or classroom, as these contexts serve as proxies for the group’s code of conduct based on behaviors reported by others. Contrasting with the descriptive, injunctive, and subjective norms that have been demonstrated to be effective in previous studies for explaining involvement in risky sexual behaviors (Baumgartner et al., 2011; Lippman & Campbell, 2014; Wilson et al., 2021), to our knowledge, collective norms have not yet been shown to influence cyberviolence behaviors, although they show promise for potential application in this field of study.

Understanding the role of social norms in shaping online risky behaviors can provide deeper insights into the mechanisms driving bystanders’ responses to NCS. The TNSB suggests that social norms will be most predictive of behaviors when the levels of other related factors are also high. Furthermore, questioning the existence of factors that could be facilitating and influencing the effects of social norms in shaping behavior is essential to understand the complex dynamics involved in NCS (Rimal et al., 2005).

### Personal Factors, Behavioral Attributes, and Contextual Factors

As argued by Rimal and Yilma (2022), the influence of social norms on behavior could be attenuated or enhanced by different factors, such as personal factors, behavioral attributes, or contextual factors. Some personal factors (i.e., intrinsic characteristics of individuals) have been identified as meaningful in terms of the dynamics of NCS. The emotional field is one of the most studied (Ortega et al., 2009). Specifically, empathy has also been associated with risky sexual behaviors online, with evidence suggesting that lower levels of empathy are associated with a higher likelihood of engaging in such behaviors (Silva et al., 2020). Furthermore, its relevance for understanding the responses of bystanders to bullying and cyberbullying has also recently been explored, with defenders exhibiting higher levels of empathy toward victims (Ma et al., 2019). Similarly, studies exploring factors facilitating bystander defender responses to forms of online and offline sexual violence have found that empathy is significantly associated with intervening in these violent contexts (Mainwaring et al., 2023a, 2023b). Although the presence or absence of empathy has been recognized as fundamental to understanding the emergence and development of violence and cyberviolence, specific research in the context of NCS remains limited. Therefore, further advances in understanding bystander responses and intentions in the context of NCS are needed.

Behavioral attributes refer to the fundamental or underlying characteristics that define the dynamics of a particular behavior (Rimal & Lapinski, 2015). Behaviors can be categorized into different classes of behavioral attributes varying in significance (Lapinski & Rimal, 2005). In the context of sexting, several studies have pointed to the significance of attributes such as moral judgments (Ricon & Dolev-Cohen, 2024) and beliefs about the behavior (Clancy et al., 2019), especially in relation to gender dynamics (Naezer & Van Oosterhout, 2021; Wilkinson et al., 2016). Notably, sexting is a practice influenced by the same gender inequalities and stereotypes that govern face-to-face situations (Burén & Lunde, 2018; Woodward et al., 2017), where the same heteronormative expectations imposed by society play a role (Villacampa, 2017).

In the context of offline sexual aggression, heteronormativity may contribute to the normalization and minimization of certain harmful behaviors, particularly those perpetrated by males against females (Yamawaki, 2007). These norms shape perceptions of blame, victimization, and responsibility, often shifting the onus onto female victims while excusing or downplaying male aggression (Endendijk et al., 2020; Zaikman & Marks, 2014). Similar dynamics are evident in the online context. For example, boys’ dissemination of intimate images is frequently dismissed as “boys being boys,” while girls are shamed and held accountable for their victimization (Naezer & Van Oosterhout, 2021; Ringrose et al., 2013).

This differentiation in societal expectations between genders is known as the sexual double standard, which grants boys greater sexual freedom while subjecting girls to social sanctions for engaging in similar behaviors (Paquette et al., 2022; Toomey et al., 2012). Given that these heteronormative beliefs are deeply embedded in cultural and social narratives, it is plausible to assume that they also shape bystanders’ responses. For instance, bystanders might be less likely to intervene in cases of NCS when victims are perceived to have violated traditional gender expectations, such as willingly engaging in sexting. However, despite the relevance of heteronormative attitudes and beliefs to involvement in NCS victimization and aggression, research on the role of bystanders is limited, so it is essential to explore the possible influence of these factors on the behavior of bystanders of this type of cyberviolence.

In addition, previous studies have emphasized the importance of contextual factors (i.e., elements external to the individual) when facing violent behavior among peers, in online contexts (Martin-Criado et al., 2021; Pöyhönen et al., 2010; Van Cleemput et al., 2014; Zhang et al., 2010). Of these, online parental supervision stands out in the literature as a relevant protective factor that can shape the attitudes and behaviors of individuals in relation to cyberviolence (Elsaesser et al., 2017; Khurana et al., 2015). Furthermore, online parental supervision has been found to reduce involvement in phenomena such as cyberbullying, in terms of both victimization and aggression (Baldry et al., 2019; Martin-Criado et al., 2021).

Given the inherent nature of NCS—which includes aggravated elements of violence, such as the violation of consent—it is particularly important to investigate how the actions of authority figures, including parents, could influence bystander behavior. However, the potential role of parental supervision in shaping bystander responses to NCS remains unexplored. Focusing research on this area could help clarify whether online parental supervision acts as a protective factor in NCS forwarding chains. For instance, it would be valuable to study whether high levels of parental supervision foster empathetic and prosocial responses among adolescents, encouraging bystanders to actively intervene or report harmful behaviors rather than remaining passive or becoming reinforcers of the abuse.

### Current Research

Applying the multidimensional approach offered by the TNSB to understand the behavior of bystanders and their intentions toward NCS would advance our understanding of this phenomenon. By reinterpreting the interactions among social norms, personal factors, behavioral attributes, and contextual factors, this approach would make it possible to combine the elements previously studied in a fragmented manner within the framework provided by the TNSB. A concise summary of the key concepts within the framework is presented in Table 1.

**Table 1:** Overview of key concepts and definitions in the study

Main categories	Subcategories	Definition
Social norms	Descriptive norms	Individuals’ perceptions of how common a behavior is within their social environment (Rimal & Real, 2005)
	Injunctive norms	Individuals’ perceptions of what their reference group believes they should do (Rimal & Real, 2005)
	Subjective norms	Individuals’ perceptions of others’ expectations regarding their engagement in a specific behavior (Hayashi & Tahmasbi, 2022)
	Collective norms	Guidelines that outline which behaviors are acceptable or unacceptable within a group (Lapinski & Rimal, 2005)
Additional factors	Empathy	The ability to understand and share the feelings of others, fostering social connection and compassion (Cuff et al., 2016)
	Heteronormative attitudes and beliefs	Beliefs and assumptions that prioritize heterosexuality as the norm (Habarth, 2015)
	Online parental supervision	Parental actions aimed at protecting their children from exposure to risky activities and online dangers (Livingstone et al., 2015)
Bystanders’ responses and intentions toward non-consensual sexting	Defensive responses	Proactive actions aimed at supporting the victim, reporting the incident, or attempting to stop the perpetrator (Mainwaring et al., 2023a; Salmivalli et al., 1998)
	Passive responses	Inaction or lack of response when witnessing an event of cyberviolence (Mainwaring et al., 2023a; Salmivalli et al., 1998)
	Reinforcing responses	Actions that support or encourage the perpetrator, such as liking, joking, or commenting positively on harmful content (Mainwaring et al., 2023a; Salmivalli et al., 1998)

Therefore, the main objective of this study is to understand which factors within the TNSB influence young people’s decision to intervene, or not, in NCS by applying the TNSB framework to explain bystander responses to this phenomenon. Specifically, the aims are to (1) examine the capacity of the fundamental constructs of the TNSB (i.e., descriptive norms, injunctive norms, subjective norms, and collective norms) to explain bystander responses and intentions in the context of the NCS; and (2) investigate whether explanatory power can be improved by including factors such as online empathy, heteronormative attitudes and beliefs, and online parental supervision, and determine the extent to which these variables explain reinforcing, defensive, and passive responses and intentions in the context of NCS.

Based on the literature described above, the following research hypotheses are proposed: (H1) Social norms indicating greater acceptance of online risky behaviors would be directly associated with reinforcing and passive bystander responses and intentions, whereas norms with lower acceptance of such risks would be directly linked to defensive responses and intentions. (H2) Including variables such as online empathy, heteronormative attitudes and beliefs, and online parental supervision significantly increases the explanatory capacity of NCS bystander response models and significantly influences the relation between social norms and bystander responses and intentions. Specifically, (H2.1) low levels of online empathy facilitate reinforcing and passive responses and intentions, whereas high levels facilitate defensive responses and intentions. (H2.2) Strong heteronormative attitudes and beliefs facilitate reinforcing responses and intentions, whereas weak ones facilitate defensive and passive responses and intentions. (H2.3) Strong perceived parental supervision facilitates defensive responses and intentions, whereas weak perceptions facilitate reinforcing and passive responses and intentions.

## Method

### Participants

A total of 2539 students from 18 schools in Andalusia, Spain, participated in this study. In terms of gender, 49.2% identified themselves as girls (n = 1245), 49.1% as boys (n = 1243), and 1.7% (n = 43) as gender diverse. Their ages ranged from 11 to 18 years (M = 14.07, SD = 1.39). These students were enrolled across different educational levels: 26.1% were in the 1st year of compulsory secondary education (n = 663), 26.5% in the 2nd year (n = 674), 24.4% in the 3rd year (n = 620), 19.1% in the 4th year (n = 486), and 3.8% were in the 1st year of High School (n = 96). From public data available on these schools, sixteen were predominantly in middle-class areas, while two were located in socio-economically disadvantaged neighborhoods.

### Procedure

Data collection was carried out using incidental sampling based on accessibility during the first semester of 2023. Principals of middle and high schools in the province of Andalusia, Spain, were contacted via institutional email and provided with an overview of the study objectives and data collection protocol. Once a school expressed interest in participating, the study was formally presented to the school’s board for review and approval. Only after obtaining this institutional approval was written informed consent requested from the parents or legal guardians of students. For data protection reasons, signed parental consents were not stored by the research team, and students without verified parental consent were not present in the classroom during data collection sessions. Data collection sessions were scheduled during school hours in coordination with school staff. In each participating class group, a member of the research team or, in some cases, a trained teacher entered the classroom and addressed the students collectively. The study’s purpose, procedures, and voluntary nature were explained in age-appropriate terms, with emphasis on confidentiality, anonymity, and the right to withdraw at any time without any academic consequences. Students were then asked to provide their individual assent if they wished to participate. Assent was indicated by signing the front page of the questionnaire.

Those who assented individually completed paper-based Spanish questionnaires that included items assessing demographic information and scales measuring key variables relevant to the study. Students who chose not to participate were escorted to a different classroom where they engaged in regular academic tasks prepared by the school. No identifying information was collected, and all responses were anonymized at the point of collection to ensure data confidentiality. Although data were collected during scheduled sessions within existing classroom groups, the total number of eligible students initially invited to participate across schools was not recorded. Due to the data protection system implemented, the research team did not have access to identifying information or comprehensive enrollment lists. Consequently, it was not possible to determine the exact size of the sampling frame or to calculate an accurate response rate.

### Measures

*Sociodemographic section*. Included questions that cover information such as age, gender, and educational center from the participants of the study.

*Non-consensual sexting involvement* was assessed through three adapted items from the Sexting Behavior and Motives Questionnaire (Del Rey et al., 2021), translated into Spanish. An introduction that includes the following brief definition of the phenomenon: “Non-consensual sexting is the act of sharing or forwarding a text message, image, or video over the Internet to one or more people, where another person appears nude or semi-nude without their consent” was included. It includes two items referring to experiences of aggression in non-consensual sexting (i.e., forwarding of girls’ content and forwarding of boys’ content) and one item related to victimization in non-consensual sexting. Each of the three selected items (e.g., “Has anyone in the last 12 months ever forwarded any of your content of this type without your permission?”; “Have you forwarded this type of content featuring girls at any time in the past 12 months?”) included five Likert-type response options ranging from “Never” (0) to “Daily” (4). (α = .75). These items constituted descriptive norms and were also utilized to calculate collective norms.

*Collective norms* (i.e., non-self-mean), following the recommendation of Sedlander and Rimal (2019), were calculated at the school level for the three NCS victimization and aggression items (i.e., descriptive norms). For each participant, their individual score on a given item was subtracted from the overall school mean for that item. The resulting value was then divided by one less than the total number of individuals in the school. This process was applied to the three items measuring victimization and aggression, resulting in separate collective norm values for each item.

*Injunctive norms* regarding online risky behaviors were assessed through three scales: friends’ injunctive norms, teachers’ injunctive norms, and families’ injunctive norms, all of those were translated into Spanish from their original versions and consist of five Likert-type items with five response options varying in the degree of agreement, ranging from 0 (Strongly Disagree) to 4 (Strongly Agree). The first scale, friends’ injunctive norms included four items developed by Sasson and Mesch (2016) and one additional item adapted to online risk behavior created by Bastiaensens et al. (2016) (e.g., “Most of my friends think it is okay to post personal data on the Internet”; “Most of my friends think it’s okay to act violently online or through mobile devices”) (α = .83). The fit indices indicated that a one-dimensional factorial solution was optimal (CFA χ^2^_SB_ = 86.67; *p* < .001; RMSEA = .07; SRMR = .04; CFI = .98; NNFI = .97).

The scale for evaluating teachers’ injunctive norms regarding online risky behaviors includes four items developed by Sasson and Mesch (2016), and one additional item adapted to online risk behavior created by Bastiaensens et al. (2016) (e.g., “My teachers believe that it is not okay to act violently online”; “My teachers recommend that I avoid posting offensive content on social media”) (α = .87). The fit indices indicated that a one-dimensional factorial solution was optimal (CFA χ^2^_SB_ = 38.22; *p* < .001; RMSEA = .05; SRMR = .02; CFI = .99; NNFI = .99).

The family’s injunctive norms regarding online risky behaviors includes four items developed by Sasson and Mesch (2016), and one additional item adapted to online risk behavior was created by Bastiaensens et al. (2016) (e.g., “My family would not approve of me acting violently online”; “My family forbids me to upload offensive posts to the networks”) (α = .89). The fit indices indicated that a one-dimensional factorial solution was optimal (CFA χ^2^_SB_ = 117.35; *p* < .001; RMSEA = .08; SRMR = .04; CFI = .98; NNFI = .97).

*Subjective norms* regarding online risky behaviors were assessed through three Likert-type items, developed by Hayashi and Tahmasbi (2022), translated into Spanish, with seven response options ranging from 0 (Strongly Disagree) to 6 (Strongly Agree) (e.g., “People who are important to me would support me in helping someone who suffers violence on the Internet”; “People who are important to me would want me to help someone experiencing online violence”) (α = .88).

Empathy toward victims of online risky behaviors, as a personal factor, was assessed through five items developed by Hayashi and Tahmasbi (2022), translated into Spanish, with seven Likert-type response options ranging from 0 (Strongly Disagree) to 6 (Strongly Agree) (e.g., “When I see someone suffering violence on the Internet, I want to help them”; “I usually feel bad when I see violence on the Internet”) (α = .91). The fit indices indicated that a one-dimensional factorial solution was optimal (CFA χ^2^_SB_ = 52.70; *p* < .001; RMSEA = .06; SRMR = .02; CFI = .99; NNFI = .98).

Heteronormative ideas and beliefs, as a behavioral attribute, were assessed through the Heteronormative Attitudes and Beliefs Scale (Habarth, 2015), translated into Spanish, which consists of eight items on normative behavior with seven response options differing in the degree of agreement, ranging from 0 (Strongly Disagree) to 6 (Strongly Agree) (e.g., “The best way to raise a child is for the mother and father to raise the child together”; “There are particular ways in which men should act and particular ways in which women should act in relationships”) (α = .75). The fit indices indicated that a one-dimensional factorial solution was optimal (CFA χ^2^_SB_ = 1068.76; *p* < .001; RMSEA = .05; SRMR = .04; CFI = .98; NNFI = .97).

Online parental supervision, as a contextual factor, was assessed using the Parental Internet Supervision Scale by Martín-Criado et al. (2021), translated into Spanish, which consists of four items with five response options differing in frequency, ranging from 0 (Never) to 4 (Always) (e.g., “My family helps me to make proper use of social networks”; “My family does things with me on the Internet [searching for information, playing games, visiting profiles…]”) (α = .73). The fit indices indicated that a one-dimensional factorial solution was optimal (CFA χ^2^_SB_ = 52.80; *p* < .001; RMSEA = .07; SRMR = .03; CFI = .99; NNFI = .97).

The *types of NCS bystanders responses and intentions* were assessed using an adapted version of the Student Spectator Behavior Scale (SBBS; Thornberg & Jungert, 2013) translated to Spanish by Álvarez-García et al. (2021). This instrument was adapted by incorporating references to NCS into the introduction part of the scale: “Now think about this academic course. Have you seen any cases of NCS? How did you respond, or do you think you would have responded?” The scale consists of 10 items, with five response options ranges in frequency from 0 (Never) to 4 (Always). Specifically, three items were used to measure reinforcing responses and intentions (e.g., “I read the rest of the comments because they were funny and amusing”; “I laughed and intervened in the comment thread to encourage the offender to keep doing it”) (α = .74), four items were used for the defensive responses and intentions (e.g., “I encouraged the victim to tell an adult person”; “I told a trusted adult or reported it on the social network”) (α = .85), and three items were used for the passive responses and intentions (e.g., “I avoid getting involved, I don’t support either one or the other”; “Nothing. I kept on doing what I was doing because it wasn’t with me”) (α = .77). The fit indices indicated that a three-dimensional factorial solution was optimal (CFA: χ^2^_SB_ = 136.60; *p* < .001; RMSEA = .04; SRMR = .05; CFI = .99; NNFI = .98).

### Analytic Strategy

CFA and reliability analysis using Cronbach’s alpha coefficients was performed to validate the adapted scales’ psychometric properties using EQS 6.4 software. Additionally, basic descriptive statistics for each study construct and a missing data analysis were performed using SPSS 29.0 software. Based on the first objective, three structural equation models (SEMs) were specified to estimate the direct effects of social norms on the three types of NCS bystander responses and intentions (reinforcing, defensive, and passive). In line with the second objective, three additional SEMs were estimated, one for each response type, incorporating online empathy, heteronormative attitudes and beliefs, and online parental supervision as additional factors. These models were built using latent variables formed by the observed variables listed in the measures section. All six models were estimated using Mplus (version 8.0), employing the robust maximum likelihood estimator (MLR), which accounts for missing data through full information maximum likelihood (FIML) under the missing at random (MAR) assumption (Enders & Bandalos, 2001), supported by a preliminary attrition analysis. To account for the nested data structure, classrooms were included as cluster variables. This was supported by intraclass correlation coefficients (ICCs) above .05, suggesting meaningful within-group dependence. Model fit was assessed using the scaled Satorra–Bentler Chi-square (χ^2^_SB_); the CFI (considered adequate if ≥ .90 and optimal if ≥ .95); and the RMSEA and the SRMR (considered adequate if ≤ .08) (Browne & Cudeck, 1992). Path coefficients were interpreted as statistically significant at *p* < .05.

#### Attrition Analysis

An attrition analysis was performed to examine potential differences between participants with complete data (57.8%, *n* = 1468) and those with at least one missing data point in any of the study variables (42.2%, *n* = 1070). The groups were compared in terms of the mean variables. Significant differences were found between the two groups based on descriptive norms (i.e., aggression experiences NCS [*t*(2305) =  − 4.65, *d* = 0.20], and victimization experiences NCS [*t*(2293) =  − 3.75, *d* = 0.16]), friends’ injunctive norms [*t*(2438) =  − 2.66, *d* = 0.11], families’ injunctive norms [*t*(2438) = 5.61, *d* = 0.23], teachers’ injunctive norms [*t*(2440) = 5.57, *d* = 0.23], subjective norms [*t*(2267) = 6.89, *d* = 0.30], collective norms [*t*(2537) =  − 7.76, *d* = 0.30], online empathy [*t*(2263) = 5.95, *d* = 0.26], heteronormative attitudes and beliefs [*t*(2432) =  − 7.05, *d* = 0.29], online parental supervision [*t*(2423) = 3.56, *d* = 0.14], reinforcing responses and intentions [*t*(2113) =  − 7.63, *d* = 0.26], defensive responses and intentions [*t*(2120) = 5.67, *d* = 0.26], and passive responses and intentions [*t*(2122) =  − 2.02, *d* = 0.09]. Despite these differences, effect sizes indicated by Cohen’s *d*, were small in all cases. Therefore, these differences were not considered meaningful for further analysis.

## Results

Descriptive analyses revealed that the predominant response type and behavioral intention was defensive (M = 2.20; SD = 1.32), followed by passive (M = 1.32; SD = 1.19) and reinforcing (M = 0.20; SD = 0.55) (Table 2).

**Table 2:** Descriptive statistics

Variables	Mean	*SD*
*Descriptive norms*		
Non-consensual forwarding (girl content)	0.12	0.50
Non-consensual forwarding (boy content)	0.09	0.43
Victim of non-consensual forwarding	0.11	0.51
*Injunctive norms*		
Friends’ injunctive norms	0.78	0.78
Families’ injunctive norms	3.28	0.91
Teachers’ injunctive norms	3.05	1.02
*Subjective norms*	4.51	1.52
*Collective norms*	0.11	0.04
*Additional factors*		
Online empathy	4.34	1.56
Heteronormative attitudes and beliefs	2.03	1.22
Online parental supervision	2.04	1.12
*Bystanders’ responses and intentions*		
Defensive response	2.20	1.32
Passive response	1.32	1.19
Reinforcing response	0.20	0.55

For friends’, families’, and teachers’ injunctive norms, descriptive norms, and parental supervision (Min = 0; Max = 4); for subjective norms, heteronormative attitudes and beliefs, and empathy (Min = 0; Max = 6)

### Study Aim 1: Examining the Fundamental Constructs of the Theory of Normative Social Behavior

**Fig. 1:**
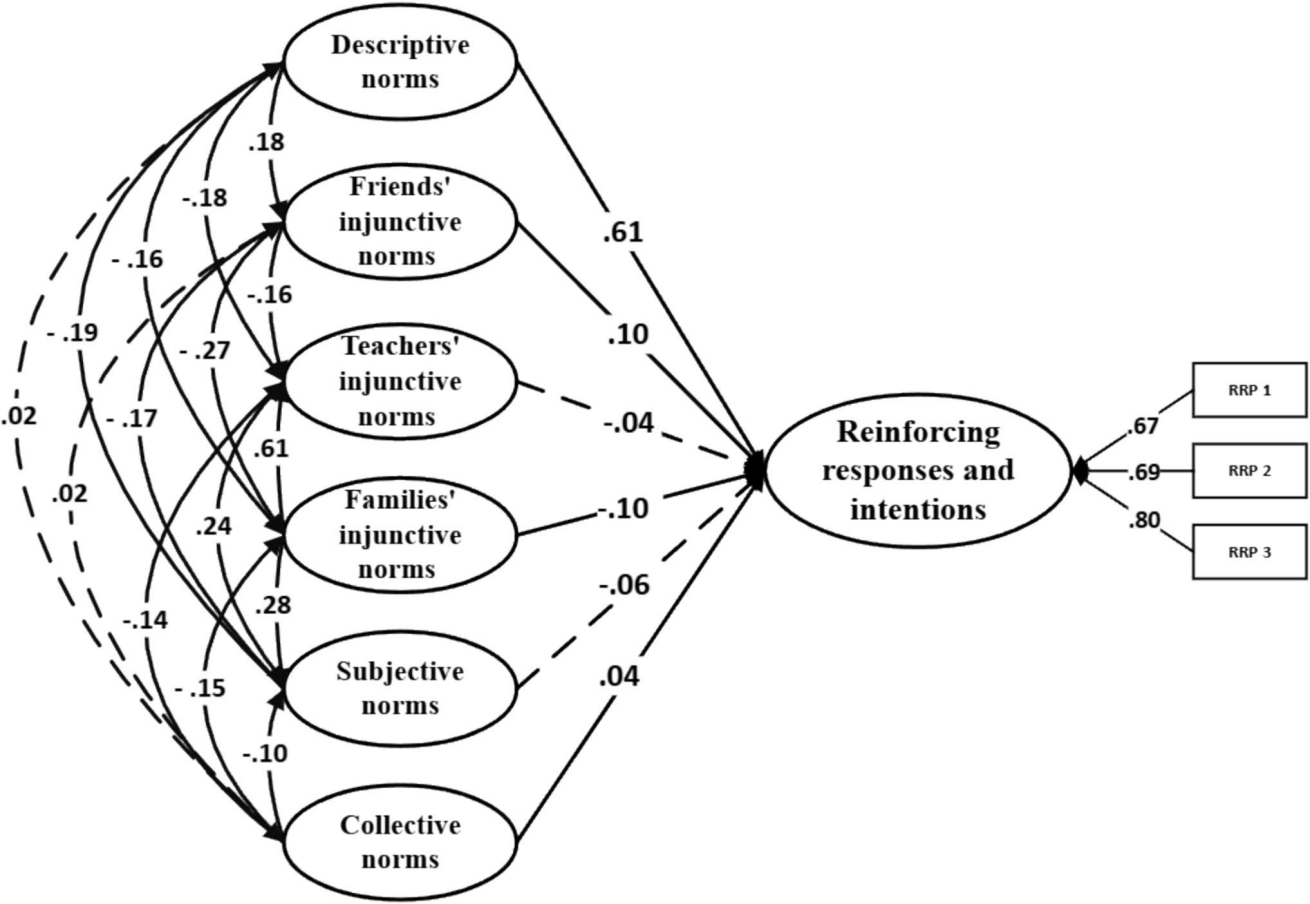
Theory of normative social behavior core model and reinforcing responses and intentions in non-consensual sexting. Note. Dashed lines denote non-significance

**Fig. 2:**
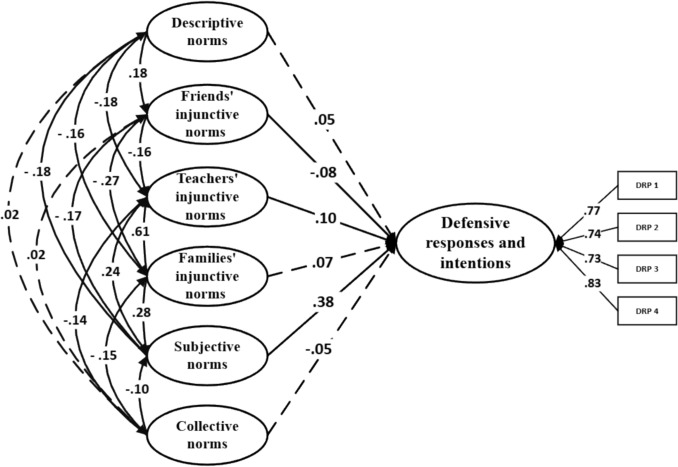
Theory of normative social behavior core model and defensive responses and intentions in non-consensual sexting. Note. Dashed lines denote non-significance

**Fig. 3:**
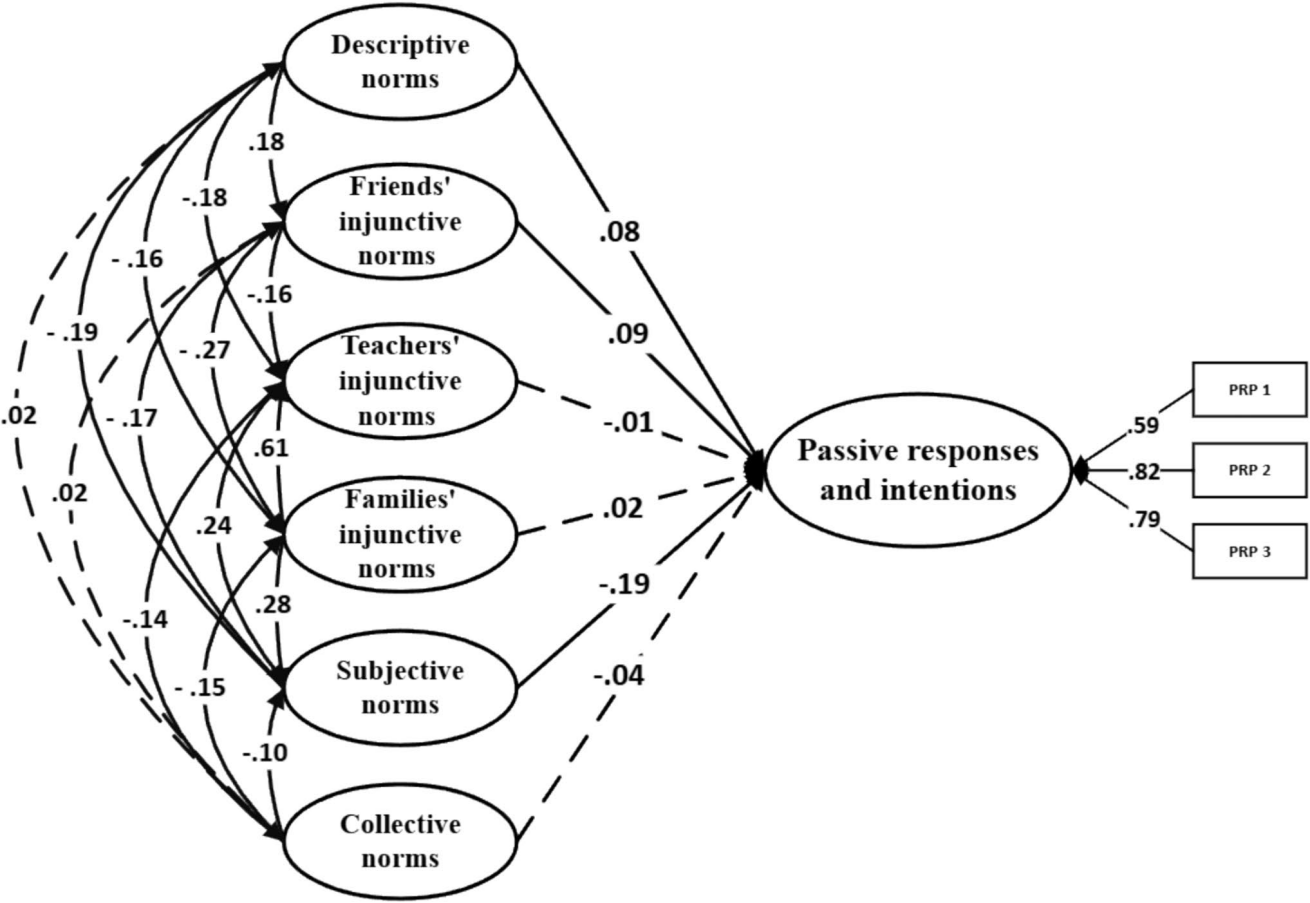
Theory of normative social behavior core model and passive responses and intentions in non-consensual sexting. Note. Dashed lines denote non-significance

The structural equation model testing direct relationships between social norms and each of the three types of bystander responses and intentions demonstrated adequate fit across all three models. Regarding reinforcing responses and intentions, the results were as follows: χ^2^_SB_ = 1448.16; *p* < .001; RMSEA = .04 [CI_90% =_ .037, .041]; SRMR = .03; CFI = .92. A total of 48.5% of the variance was explained. Regarding defensive responses and intentions, model fit was also acceptable: χ^2^ SB = 1719.31; *p* < .001; RMSEA = .04 [CI_90%_ = .039, .043]; SRMR = .03; CFI = .92. The model explained 22.7% of the variance. Finally, for passive responses and intentions, the model showed a similarly good fit: χ^2^ SB = 1558.27; p < .001; RMSEA = .04 [CI_90%_ = .038, .042]; SRMR = .03; CFI = .92; with 6.6% of the variance explained. The standardized β coefficients revealed several significant associations across the models. In the reinforcing responses and intentions model, friends’ injunctive norms (β = .10;* p* < .001), families’ injunctive norms (β = – .10;* p* = .032), descriptive norms (β = .61;* p* < .001), and collective norms (β = .04;* p* = .039) showed statistically significant effects (Fig. 1). In the defensive responses and intentions model, subjective norms (β = .38; *p* < .001), friends’ injunctive norms (β = –.08;* p* = .004), and teachers’ injunctive norms (β = .10;* p* = .002) emerged as significant variables (Fig. 2). Lastly, in the passive responses and intentions model, subjective norms (β = –.19; *p* < .001), friends’ injunctive norms (β = .09;* p* = .002) and descriptive norms (β = .08;* p* = .014) were found to be significant (Fig. 3).

### Study Aim 2: Improving the Model by Including Behavioral Attributes, Personal, and Contextual Factors

The structural equation models incorporating online empathy, heteronormative ideas and beliefs, and online parental supervision showed a good fit across all three types of bystander responses. For reinforcing responses and intentions, model fit indices were as follows: χ^2^ SB = 3895.00; *p* < .001; RMSEA = .03 [CI_90%_ = .036, .038]; SRMR = .03; CFI = .90. The model accounted for 50.0% of the variance in reinforcing responses and intentions. Regarding defensive responses and intentions, the model demonstrated similar fit: χ^2^ SB = 4195.20; *p* < .001; RMSEA = .03 [CI_90%_ = .037, .039]; SRMR = .03; CFI = .90. A total of 30.2% of the variance in defensive responses and intentions was explained. Finally, for passive responses and intentions, the model also showed adequate fit: χ^2^ SB = 4044.85; *p* < .001; RMSEA = .03 [CI_90%_ = .037, .039]; SRMR = .04; CFI = .90. The model explained 11.0% of the variance in passive responses and intentions.

**Fig. 4:**
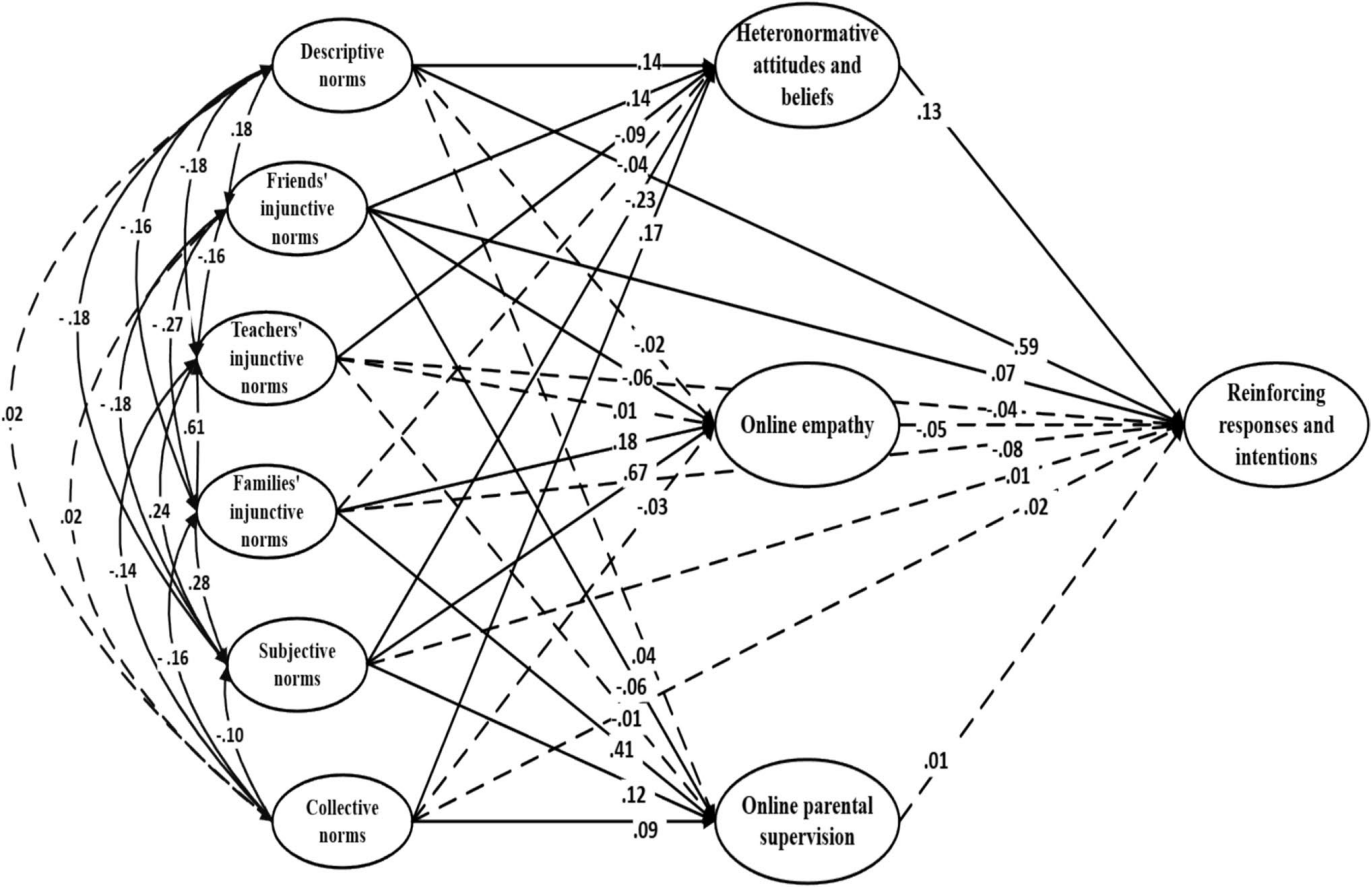
Theory of normative social behavior model (with additional variables) applied to reinforcing responses and intentions. Note. Dashed lines denote non-significance

**Fig. 5:**
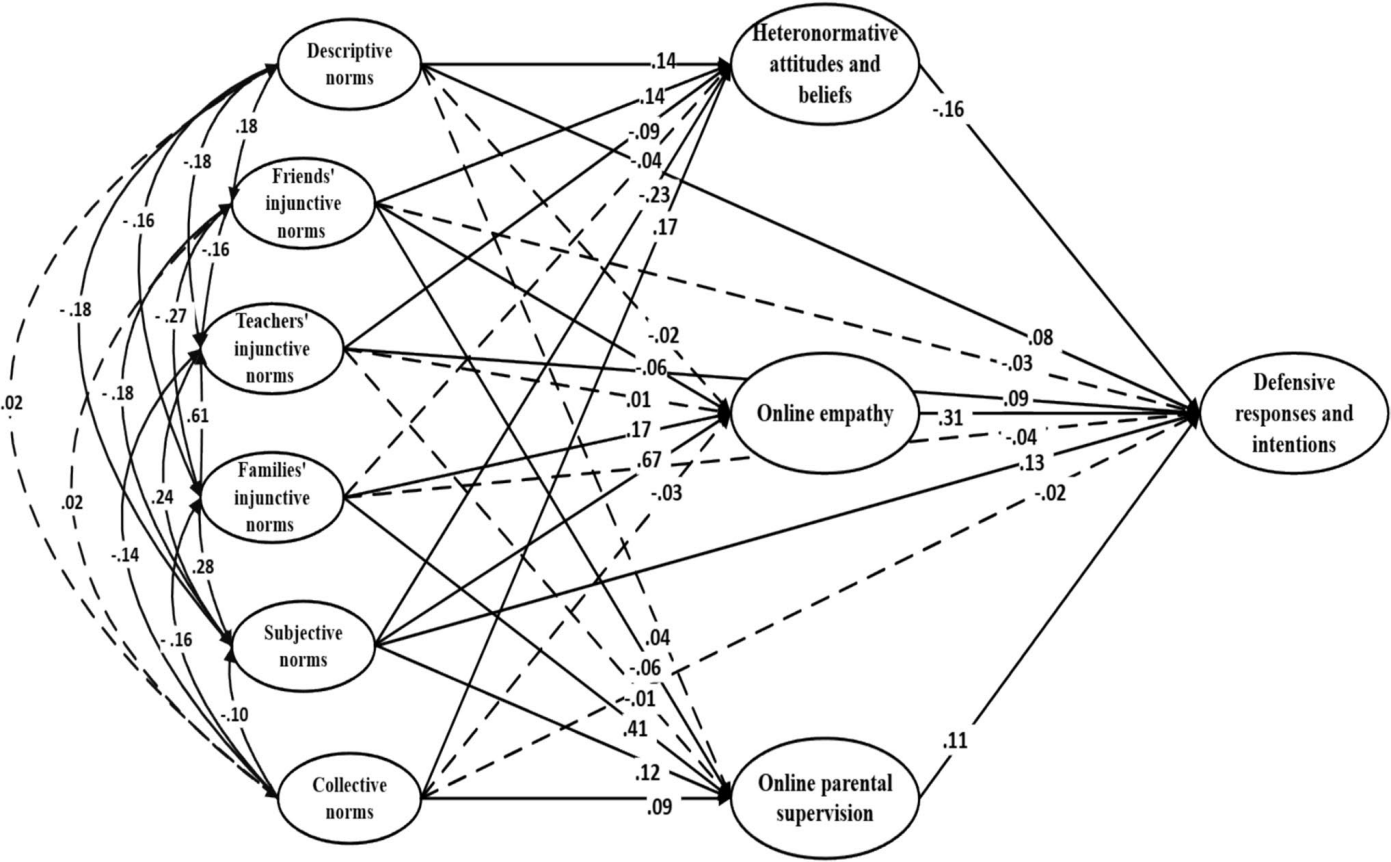
Theory of normative social behavior model (with additional variables) applied to defensive responses and intentions. Note. Dashed lines denote non-significance

**Fig. 6:**
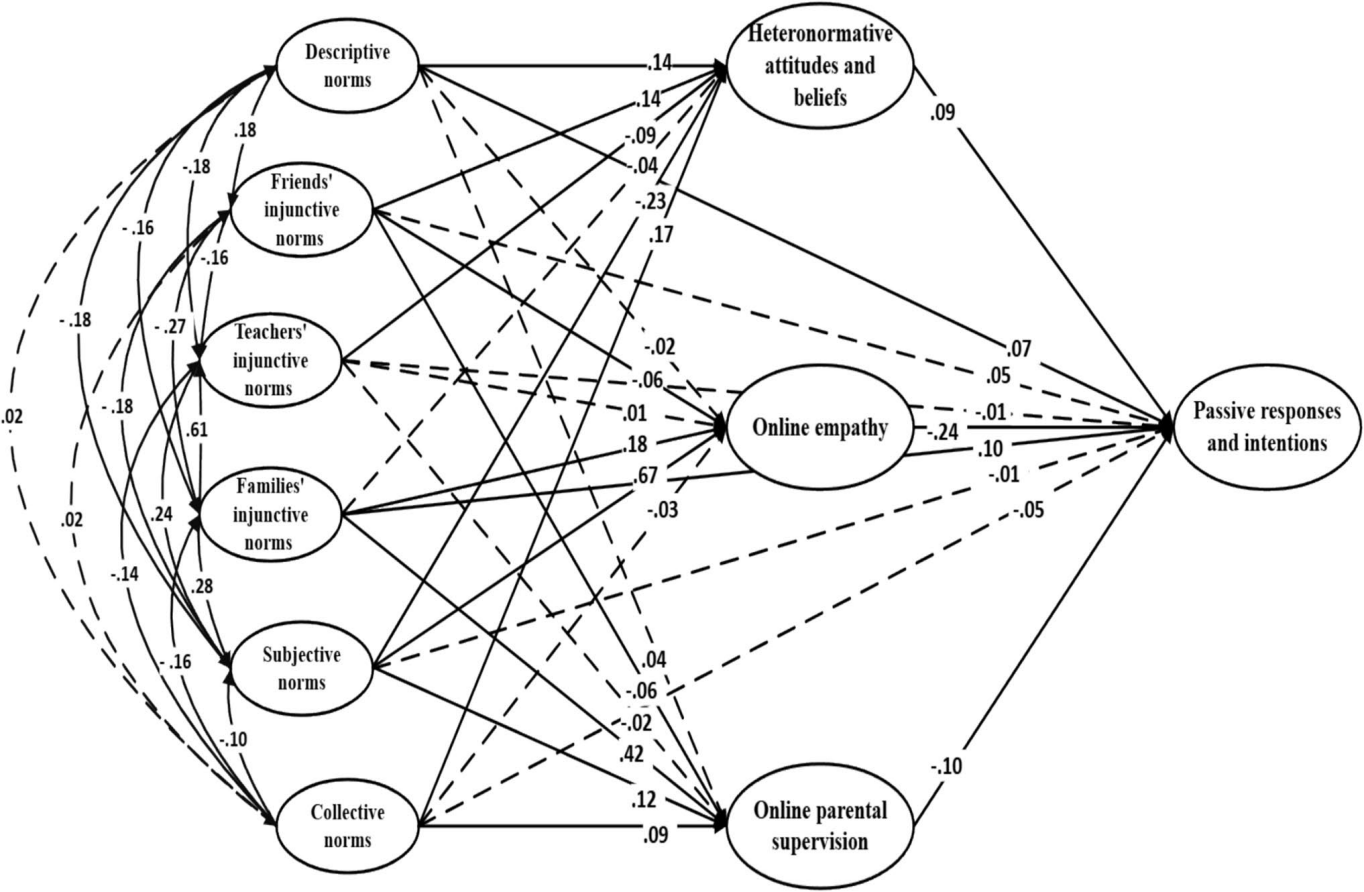
Theory of normative social behavior model (with additional variables) applied to passive responses and intentions. Note. Dashed lines denote non-significance

The direct effects of the three added variables were examined in relation to the reinforcing (Fig. 4), defensive (Fig. 5), and passive (Fig. 6) response types. Online empathy was significantly associated with both defensive and passive bystander responses. In the defensive model, it showed a positive effect (β = .31; *p* < .001), while in the passive model, it had a negative effect on this type of response (β = -.24; *p* < .001). Heteronormative attitudes and beliefs were directly associated with all three types of responses. In the reinforcing model, they had a positive effect (β = .13, *p* = .001), and similarly, a positive association was found in the passive model (β = .09, *p* = .018). Conversely, in the defensive model, the effect was negative (β = –.16, *p* < .001).

Lastly, online parental supervision was directly related to defending and passive bystander models. Specifically, it had a positive effect in the defensive model (β = .11, *p* < .001), and a negative effect in the passive model (β = –.10, *p* = .008).

## Discussion

NCS involves the unauthorized dissemination of erotic-sexual content that constitutes yet another form of sexual violence with psychological, social, and legal ramifications (Judge, 2012; Wachs et al., 2021; Walker & Sleath, 2017). Considering the importance of bystander engagement in the dynamics of this type of cyberviolence, this study mainly sought to understand which factors within the TNSB influence adolescents’ decisions to intervene or refrain from intervening in NCS as reinforcing, defensive, or passive bystanders. The roles of empathy, heteronormative attitudes and beliefs, and parental supervision in the three types of responses were also explored in an effort to improve the original model (Rimal & Real, 2005).

This study represents one of the first efforts to analyze bystander behavior in the context of NCS. Descriptive analyses revealed that defensive responses (or intention to perform them) were the most common in NCS situations, followed by passive and reinforcing behaviors. These results are aligned with previous research on bystander responses to analogous phenomena, such as cyberbullying (González‐Cabrera et al., 2019; Panumaporn et al., 2020; Schultze-Krumbholz et al., 2018), indicating a propensity among adolescents and youths to predominantly engage in prosocial actions in situations of violence, such as offering support and aid to victims.

Regarding the first aim of this study, the results indicated that the core constructs of the TNSB (injunctive norms, subjective norms, descriptive norms, and collective norms) serve as an effective framework not only for understanding involvement in peer violence (Lokot et al., 2020; Neville, 2014; Reynolds-Tylus et al., 2019; Yarnell et al., 2014), but also bystander reactions or intentions specifically to NCS as an emerging form of cyberviolence. The explanatory power of the TNSB model was strongest for reinforcing responses and intentions, followed by defensive and, to a lesser extent, passive responses.

Contrary to what was expected in H1, not all components of the TNSB explain bystander behavior and intentions; their relevance depends on the type of response. More specifically, subjective norms were significant predictors of defensive and passive responses, while friends’ injunctive norms were associated with all three types of responses. Teachers’ injunctive norms were positively linked to defensive responses, whereas families’ injunctive norms were negatively associated with reinforcing responses. Descriptive norms were relevant to both reinforcing and passive responses, while collective norms were associated exclusively with reinforcing responses. However, the direction of these associations was consistent with our expectations: social norms reflecting lower acceptance of online risks were positively associated with defensive responses and intentions, whereas norms suggesting greater acceptance of online risks were linked to reinforcing and passive responses.

These findings reinforce the idea that adolescents are more likely to engage in defensive responses when they perceive that authority figures expect them to behave prosocially in digital contexts. Conversely, they opt for passive and reinforcing responses if they believe that significant others prefer not to intervene in NCS. In this line, subjective norms emerged as a particularly influential factor in understanding defensive and passive responses. These behavioral patterns may be linked to the adolescents’ need to meet social expectations and maintain positive relationships with their important referents, as well as to the fear of potential negative consequences such as sanctions or punishment (Ajzen, 1991; Barrense-Dias et al., 2024; Cialdini & Goldstein, 2004; Rimal & Real, 2005).

Further supporting this interpretation, the findings point to the role of adult figures. Teachers’ injunctive norms were positively associated with defensive responses, suggesting that school authority can promote prosocial online behavior when expectations are clear (Salmivalli, 2014). Conversely, families’ injunctive norms were negatively related to reinforcing responses, indicating that strong parental disapproval of online risks may discourage adolescents from supporting aggressive behavior (Bullo & Schulz, 2022). These results underscore the importance of adult guidance in shaping adolescents’ digital decisions.

The role of friends’ injunctive norms also emerged as a central factor in bystander behavior. It was found that adolescents may engage in reinforcing or passive behaviors and intentions toward NCS if they perceive that their friends approve risky online behaviors. In contrast, they tend to adopt defensive responses when they perceive disapproval toward such behaviors. These results align with numerous research studies emphasizing peer influence as a determining factor in adolescent decision-making (Baumgartner et al., 2011; Dolev-Cohen, 2023; Mainwaring et al., 2023b; Walrave et al., 2014). Previous studies have already highlighted that adolescents’ intention to engage in sexting is associated with the extent to which they perceive that their peers approve of this phenomenon (Van Ouytsel et al., 2017a). Thus, participation as a bystander in NCS may be driven by how adolescents perceive this practice would affect their reputation among their peers (Walrave et al., 2015). This underscores the need to consider the dynamics of peer relationships when developing preventive programs to enhance defensive responses in cyberviolence phenomena such as NCS.

Descriptive norms, previously identified as predictors of adolescents’ engagement in risky sexual behaviors online (Baumgartner et al., 2011), were found in this study to be significantly associated with both reinforcing and passive bystander responses and intentions in the context of NCS. Notably, they showed the strongest positive association with reinforcing behaviors. In contrast, collective norms were associated only with reinforcing responses. The normalization of violence may explain these findings. If adolescents perceive that NCS is perpetrated or tolerated by those around them, they may perceive these behaviors as normal (Gámez-Guadix et al., 2017; Strassberg et al., 2013). Thus, although they may not always intend to engage in certain behaviors, they may be willing to do so in certain situations, for example, if they believe that most of their friends do (Gerrard et al., 2008; Walrave et al., 2015). This perception of peer consensus may shape their attitudes and responses, increasing the likelihood of reinforcing or passively accepting online aggression rather than intervening to stop it.

This research also aimed to determine whether we could improve the model by including some personal factors, behavioral attributes, and contextual factors based on the proposal of Rimal and Yilma (2022). The results revealed that the inclusion of variables such as online empathy, heteronormative attitudes and beliefs, and online parental supervision affects the variance in bystanders' responses and intentions, improving the explanatory power of the models for the three types of bystander responses and intentions, which confirms H2. This suggests that the presence of these variables is crucial for understanding bystander behavior in cases of NCS.

Specifically, online empathy was particularly relevant to defensive and passive responses, highlighting that defensive responses to NCS situations are associated with elevated levels of empathy and partially confirming H2.1. This finding aligns with the growing literature on factors predicting adolescents’ positive intervention in aggression and microaggressions in real-world and virtual contexts (Aliberti et al., 2023; Mainwaring et al., 2024; Marks et al., 2024; Zhao et al., 2023). This finding may be explained by the fact that high levels of empathy allow adolescents to connect emotionally with the experiences of victims, which motivates them to intervene and come to their defense.

Heteronormative attitudes and beliefs emerged as significant predictors across all three types of bystander responses and intentions, fully confirming H2.2. Specifically, weak heteronormative attitudes and beliefs facilitate a defensive response, whereas strong ones facilitate reinforcing and passive responses. These findings suggest that adolescents who strongly adhere to traditional gender and sexual norms may hold more biased or prejudiced views about the implications of NCS, potentially making them more likely to support or ignore such behavior. This may reflect an internalization of idealized or socially imposed roles, where reinforcing aggression or remaining passive aligns with stereotypical expectations around masculinity, femininity, or sexuality (Habarth, 2015; Mishna et al., 2023).

Finally, findings regarding online parental supervision suggest that unsupervised bystanders tend to adopt passive responses to NCS situations, whereas those who perceive high parental supervision tend to react defensively, partially confirming H2.3. These findings are consistent with a number of studies emphasizing the role of parental supervision in adolescent development and decision-making (Corcoran et al., 2022; Dolev-Cohen, 2023; Martin-Criado et al., 2021; Panumaporn et al., 2020; Pistoni et al., 2023). Therefore, it is critical to establish clear boundaries and rules about the use of mobile devices, and to foster safe and supportive environments and open communication with adolescents. These measures not only help to mitigate the risks associated with NCS but also promote defensive attitudes in adolescents, which can help to reduce aggressive online behaviors (Jiang et al., 2016).

### Practical Implications

Beyond representing a significant advance in understanding the role of bystanders in NCS, this study could also help lay the groundwork for prevention strategies targeting bystanders of cyberviolence, particularly NCS. While our findings indicate that adolescents tend to adopt or opt for defensive responses to NCS, a notable proportion would engage in reinforcing or passive behaviors, albeit to a lesser extent. Consequently, given the protagonism of these actors in the phenomenon, the implementation of intervention programs targeting bystanders should be a current priority.

Consistent with evidence from other forms of sexual violence, bystander programs have demonstrated significant effects in enhancing bystander efficacy and increasing participants’ rates of bystander intervention, especially when targeted at students at younger ages (Kettrey & Marx, 2019a, 2019b). Building upon these established approaches could help adapt and refine interventions for addressing NCS. Furthermore, those bystander intervention programs should also incorporate established models, such as the five-step process for effective bystander intervention (Darley & Latane, 1968), since it has been previously suggested that this could increase the efficacy of such programs, ensuring that bystanders are equipped to intervene appropriately and effectively (Burn, 2009).

Specifically, our study further emphasizes the crucial role of social norms in shaping bystander behavior. Subjective and injunctive norms significantly influence whether adolescents feel socially encouraged or discouraged from intervening, or even from considering the possibility of intervention. Therefore, educational interventions should prioritize redefining group norms, actively challenging the normalization of NCS, and promoting prosocial expectations. For example, previous cyberbullying and sexting intervention programs have employed strategies such as highlighting how certain behaviors do not lead to greater social integration or examining existing norms on social media and discussing their impact, all of which have proven effective (Del Rey et al., 2018). Schools can build on these dynamics, but targeting bystanders instead, to foster environments where defending against NCS becomes a socially valued behavior.

In our study, online empathy emerged as a pivotal factor related to bystanders’ responses to NCS, especially in defensive responses. Therefore, school-based programs that foster empathy among adolescents may play a transformative role in addressing NCS. As has been demonstrated with other forms of cyberviolence, this strategy could help cultivate a culture of prosocial behavior, while also empowering students to act defensively (Garandeau et al., 2023; Mesurado et al., 2019). In parallel, the influence of heteronormative beliefs across all response types underscores the importance of addressing gender norms and stereotypes in school-based interventions. Initiatives incorporating discussions around consent, equality, and gender sensitivity may help dismantle prejudiced beliefs that contribute to harmful online behavior (Agnew, 2021). Finally, findings regarding parental supervision suggest the need for schools and families to collaborate in establishing clear guidelines for digital behavior and maintaining open communication about online risks. Schools can further support this dynamic by providing resources and training for families, creating a unified strategy to ensure adolescents feel guided and empowered to act responsibly in the face of NCS.

### Limitations and Directions for Future Research

Despite the significant contributions of this study, several limitations should be acknowledged. First, the cross-sectional design of the study limits our ability to infer causality, and the directionality of the relationships observed. Second, future research should incorporate instruments that measure injunctive norms from a bystander intervention perspective, as well as scales that more accurately assess descriptive norms related to the perceived prevalence of peer intervention toward NCS. This approach would enhance the explanatory power of the findings by providing a more precise and comprehensive understanding of bystander behaviors in the context of NCS. Additionally, a deeper exploration of potential mediators or moderators, through an attribute-centered approach, could enhance future studies on the TNSB. Although this study contextualized bystander behaviors and intentions within the “current academic year” (i.e., during 2022–2023), this specific time frame may have influenced participants’ recollections or interpretations of events. Future research could explore bystander behaviors across a broader range of contexts and time periods to enhance the generalizability of the findings. Moreover, due to the data protection system implemented during recruitment, the total number of students who were initially eligible or invited to participate was not recorded. Consequently, it was not possible to calculate a response rate or to determine whether systematic differences exist between those who chose to participate and those who did not, limiting the assessment of potential nonresponse bias. In addition, no data on participants’ race or ethnicity were collected due to ethical constraints. Finally, our study combined the assessment of real and hypothetical bystander behaviors, which may have introduced potential biases such as socially desirable responding. This should be considered when interpreting the results.

### Conclusions

This research confirms that the TNSB constitutes a solid framework for explaining bystander behavior and intentions in NCS. Specifically, the study highlights the importance of social norms, personal factors, behavioral attributes, and contextual factors in understanding how bystanders respond to NCS. While the influence of social norms varied by response type, subjective norms, descriptive norms and friends’ injunctive norms were the most consistent predictors. Incorporating additional factors such as online empathy, heteronormative beliefs, and parental supervision, improved the explanatory power of all three models. Empathy encouraged defensive responses and reduced passivity; heteronormative beliefs predicted all three response types; and parental supervision was linked to more defensive and less passive behavior. These results highlight the importance of addressing both social norms and individual and contextual factors in developing prevention strategies aimed at fostering prosocial responses to NCS among adolescents.

## Data Availability

The data and materials used in this research are available to the scientific community upon request.

## References

[CR1] Agnew, E. (2021). Sexting among young people: Towards a gender-sensitive approach. *International Journal of Children’s Rights,**29*(1), 3–30. 10.1163/15718182-28040010

[CR2] Ajzen, I. (1991). The theory of planned behavior. *Organizational Behavior and Human Decision Processes,**50*(2), 179–211. 10.1016/0749-5978(91)90020-T

[CR3] Aliberti, M., Jenkins, L., & Monk, M. (2023). Predictors of cyberbystander intervention among adolescents. *Journal of Interpersonal Violence,**38*(9–10), 6454–6479. 10.1177/08862605221132784 36416484 10.1177/08862605221132784

[CR4] Álvarez-García, D., Thornberg, R., & Suárez-García, Z. (2021). Validation of a scale for assessing bystander responses in bullying. *Psicothema,**33*(4), 623–630. 10.7334/psicothema2021.140 34668478 10.7334/psicothema2021.140

[CR5] Baldry, A. C., Sorrentino, A., & Farrington, D. P. (2019). Cyberbullying and cybervictimization versus parental supervision, monitoring and control of adolescents’ online activities. *Children and Youth Services Review,**96*, 302–307. 10.1016/j.childyouth.2018.11.058

[CR6] Barrense-Dias, Y., Akre, C., Auderset, D., Leeners, B., Morselli, D., & Surís, J.-C. (2020). Non-consensual sexting: Characteristics and motives of youths who share received-intimate content without consent. *Sexual Health,**17*(3), 270–278. 10.1071/SH19201 32594970 10.1071/SH19201

[CR7] Barrense-Dias, Y., Suris, J.-C., & Chok, L. (2024). “Maybe they don’t necessarily realize the damage they can do…”: A qualitative study on bystanders to non-consensual forwarding of nudes among adolescents in Switzerland. *International Journal of Bullying Prevention*, *7*, 623–631. 10.1007/s42380-024-00249-2

[CR8] Barroso, R., Marinho, A. R., Figueiredo, P., Ramião, E., & Silva, A. S. (2023). Consensual and non-consensual sexting behaviors in adolescence: A systematic review. *Adolescent Research Review,**8*(1), 1–20. 10.1007/s40894-022-00199-0

[CR9] Bastiaensens, S., Pabian, S., Vandebosch, H., Poels, K., Van Cleemput, K., DeSmet, A., & De Bourdeaudhuij, I. (2016). From normative influence to social pressure: How relevant others affect whether bystanders join in cyberbullying. *Social Development,**25*(1), 193–211. 10.1111/sode.12134

[CR10] Baumgartner, S. E., Valkenburg, P. M., & Peter, J. (2011). The influence of descriptive and injunctive peer norms on adolescents’ risky sexual online behavior. *Cyberpsychology, Behavior, and Social Networking,**14*(12), 753–758. 10.1089/cyber.2010.0510 22017408 10.1089/cyber.2010.0510

[CR11] Brighi, A., Amadori, A., Summerer, K., & Menin, D. (2023). Prevalence and risk factors for nonconsensual distribution of intimate images among Italian young adults: Implications for prevention and intervention. *International Journal of Clinical and Health Psychology,**23*(4), Article 100414. 10.1016/j.ijchp.2023.100414 37772270 10.1016/j.ijchp.2023.100414 PMC10523183

[CR12] Browne, M. W., & Cudeck, R. (1992). Alternative ways of assessing model fit. *Sociological Methods & Research,**21*(2), 230–258. 10.1177/0049124192021002005

[CR13] Bullo, A., & Schulz, P. J. (2022). Do peer and parental norms influence media content-induced cyber aggression? *Computers in Human Behavior,**129*, Article 107136. 10.1016/j.chb.2021.107136

[CR14] Burén, J., & Lunde, C. (2018). Sexting among adolescents: A nuanced and gendered online challenge for young people. *Computers in Human Behavior,**85*, 210–217. 10.1016/j.chb.2018.02.003

[CR15] Burn, S. M. (2009). A situational model of sexual assault prevention through bystander intervention. *Sex Roles,**60*(11–12), 779–792. 10.1007/s11199-008-9581-5

[CR16] Carcioppolo, N., & Jensen, J. D. (2012). Perceived historical drinking norms and current drinking behavior: Using the theory of normative social behavior as a framework for assessment. *Health Communication,**27*(8), 766–775. 10.1080/10410236.2011.640973 22292928 10.1080/10410236.2011.640973

[CR17] Chung, A., & Rimal, R. N. (2016). Social norms: A review. *Review of Communication Research,**4*, 1–28. 10.12840/issn.2255-4165.2016.04.01.008

[CR18] Cialdini, R. B., & Goldstein, N. J. (2004). Social influence: Compliance and conformity. *Annual Review of Psychology,**55*(1), 591–621. 10.1146/annurev.psych.55.090902.142015 14744228 10.1146/annurev.psych.55.090902.142015

[CR19] Clancy, E. M., Klettke, B., & Hallford, D. J. (2019). The dark side of sexting: Factors predicting the dissemination of sexts. *Computers in Human Behavior,**92*, 266–272. 10.1016/j.chb.2018.11.023

[CR20] Corcoran, E., Doty, J., Wisniewski, P., & Gabrielli, J. (2022). Youth sexting and associations with parental media mediation. *Computers in Human Behavior,**132*, Article 107263. 10.1016/j.chb.2022.107263

[CR21] Costello, M., Hawdon, J., Reichelmann, A. V., Oksanen, A., Blaya, C., Llorent, V. J., Räsänen, P., & Zych, I. (2023). Defending others online: The influence of observing formal and informal social control on one’s willingness to defend cyberhate victims. *International Journal of Environmental Research and Public Health,**20*(15), 6506. 10.3390/ijerph20156506 37569046 10.3390/ijerph20156506 PMC10419263

[CR22] Cuff, B. M. P., Brown, S. J., Taylor, L., & Howat, D. J. (2016). Empathy: A review of the concept. *Emotion Review,**8*(2), 144–153. 10.1177/1754073914558466

[CR23] Darley, J. M., & Latané, B. (1968). Bystander intervention in emergencies: Diffusion of responsibility. *Journal of Personality and Social Psychology,**8*(4, Pt.1), 377–383. 10.1037/h0025589 5645600 10.1037/h0025589

[CR24] Del Rey, R., Mora-Merchán, J.-A., Casas, J.-A., Ortega-Ruiz, R., & Elipe, P. (2018). “Asegúrate” program: Effects on cyber-aggression and its risk factors. *Comunicar,**26*(56), 39–48. 10.3916/C56-2018-04

[CR25] Del Rey, R., Ojeda, M., & Casas, J. A. (2021). Validation of the Sexting Behavior and Motives Questionnaire (SBM-Q). *Psicothema*, *37*, 287–295. 10.7334/psicothema2020.207 33879302 10.7334/psicothema2020.207

[CR26] Dodaj, A., & Sesar, K. (2024). Sexting coercion within romantic context: A test of Akers’ social learning theory. *Journal of Sexual Aggression*, *30*, 197–210. 10.1080/13552600.2023.2182377

[CR27] Dodaj, A., Sesar, K., Bošnjak, L., & Vučić, M. (2023). Theory of planned behaviour and sexting intention of college students. *Emerging Adulthood,**12*(2), 163–174. 10.1177/21676968231208343

[CR28] Dolev-Cohen, M. (2023). The association between sexting motives and behavior as a function of parental and peers’ role. *Computers in Human Behavior,**147*, Article 107861. 10.1016/j.chb.2023.107861

[CR29] Durán, M., & Rodríguez-Domínguez, C. (2020). Social perception of situations of sexual cyberviolence: The role of sexist attitudes and the victim’s transgression of gender roles / Percepción social de situaciones de ciberviolencia sexual: El rol de las actitudes sexistas y la transgresión de rol de género de la víctima. *International Journal of Social Psychology,**35*(1), 148–174. 10.1080/02134748.2019.1682295

[CR30] Elsaesser, C., Russell, B., Ohannessian, C. M., & Patton, D. (2017). Parenting in a digital age: A review of parents’ role in preventing adolescent cyberbullying. *Aggression and Violent Behavior,**35*, 62–72. 10.1016/j.avb.2017.06.004

[CR31] Endendijk, J. J., Van Baar, A. L., & Deković, M. (2020). He is a stud, she is a slut! A meta-analysis on the continued existence of sexual double standards. *Personality and Social Psychology Review,**24*(2), 163–190. 10.1177/1088868319891310 31880971 10.1177/1088868319891310 PMC7153231

[CR32] Enders, C., & Bandalos, D. (2001). The relative performance of full information maximum likelihood estimation for missing data in structural equation models. *Structural Equation Modeling: A Multidisciplinary Journal,**8*(3), 430–457. 10.1207/S15328007SEM0803_5

[CR33] Gámez-Guadix, M., de Santisteban, P., & Resett, S. (2017). Sexting among Spanish adolescents: Prevalence and personality profiles. *Psicothema*, *29*, 29–34. 10.7334/psicothema2016.222 28126055 10.7334/psicothema2016.222

[CR34] Ganson, K. T., O’Connor, C., Nagata, J. M., Testa, A., Jackson, D. B., Pang, N., & Mishna, F. (2024). Associations between receiving non-consensual image and video sexts and average sleep duration among adolescents and young adults. *Sexual Health*, *21*. 10.1071/SH23202 38626204 10.1071/SH23202

[CR35] Garandeau, C. F., Turunen, T., Saarento-Zaprudin, S., & Salmivalli, C. (2023). Effects of the KiVa anti-bullying program on defending behavior: Investigating individual-level mechanisms of change. *Journal of School Psychology,**99*, Article 101226. 10.1016/j.jsp.2023.101226 37507180 10.1016/j.jsp.2023.101226

[CR36] Gerrard, M., Gibbons, F. X., Houlihan, A. E., Stock, M. L., & Pomery, E. A. (2008). A dual-process approach to health risk decision making: The prototype willingness model. *Developmental Review,**28*(1), 29–61. 10.1016/j.dr.2007.10.001

[CR37] González-Cabrera, J. M., León-Mejía, A., Machimbarrena, J. M., Balea, A., & Calvete, E. (2019). Psychometric properties of the cyberbullying triangulation questionnaire: A prevalence analysis through seven roles. *Scandinavian Journal of Psychology,**60*(2), 160–168. 10.1111/sjop.12518 30690725 10.1111/sjop.12518

[CR38] Habarth, J. M. (2015). Development of the heteronormative attitudes and beliefs scale. *Psychology & Sexuality,**6*(2), 166–188. 10.1080/19419899.2013.876444

[CR39] Harder, S. K. (2021). The emotional bystander: Sexting and image-based sexual abuse among young adults. *Journal of Youth Studies,**24*(5), 655–669. 10.1080/13676261.2020.1757631

[CR40] Harlow, A. F., Willis, S. K., Smith, M. L., & Rothman, E. F. (2021). Bystander prevention for sexual violence: #HowIWillChange and gaps in Twitter discourse. *Journal of Interpersonal Violence,**36*(11–12), NP5753–NP5771. 10.1177/0886260518808854 30379107 10.1177/0886260518808854

[CR41] Hayashi, Y., & Tahmasbi, N. (2022). Psychological predictors of bystanders’ intention to help cyberbullying victims among college students: An application of theory of planned behavior. *Journal of Interpersonal Violence,**37*(13–14), NP11333–NP11357. 10.1177/0886260521992158 33554727 10.1177/0886260521992158

[CR42] Hollá, K., Jedličková, P., & Seidler, P. (2018). Sexting and motives for sexting among adolescents. *Ad Alta: Journal of Interdisciplinary Research*, *8*(2), 10.31299/ksi.32.2.3.

[CR43] Jacobs, K. (2004). Pornography in small places and other spaces. *Cultural Studies,**18*(1), 67–83. 10.1080/0950238042000181610

[CR44] Jiang, Y., Yu, C., Zhang, W., Bao, Z., & Zhu, J. (2016). Peer victimization and substance use in early adolescence: Influences of deviant peer affiliation and parental knowledge. *Journal of Child and Family Studies,**25*(7), 2130–2140. 10.1007/s10826-016-0403-z

[CR45] Judge, A. M. (2012). Sexting” among U.S. adolescents: Psychological and legal perspectives. *Harvard Review of Psychiatry,**20*(2), 86–96. 10.3109/10673229.2012.677360 22512742 10.3109/10673229.2012.677360

[CR46] Kettrey, H. H., & Marx, R. A. (2019a). Does the gendered approach of bystander programs matter in the prevention of sexual assault among adolescents and college students? A systematic review and meta-analysis. *Archives of Sexual Behavior,**48*(7), 2037–2053. 10.1007/s10508-019-01503-1 31292784 10.1007/s10508-019-01503-1

[CR47] Kettrey, H. H., & Marx, R. A. (2019b). The effects of bystander programs on the prevention of sexual assault across the college years: A systematic review and meta-analysis. *Journal of Youth and Adolescence,**48*(2), 212–227. 10.1007/s10964-018-0927-1 30264210 10.1007/s10964-018-0927-1

[CR48] Khurana, A., Bleakley, A., Jordan, A. B., & Romer, D. (2015). The protective effects of parental monitoring and internet restriction on adolescents’ risk of online harassment. *Journal of Youth and Adolescence,**44*(5), 1039–1047. 10.1007/s10964-014-0242-4 25504217 10.1007/s10964-014-0242-4

[CR49] Klettke, B., Hallford, D. J., & Mellor, D. J. (2014). Sexting prevalence and correlates: A systematic literature review. *Clinical Psychology Review,**34*(1), 44–53. 10.1016/j.cpr.2013.10.007 24370714 10.1016/j.cpr.2013.10.007

[CR50] Krebbekx, W. (2024). A tale of girl-sends-nudes-to-boy? Unscripting sexting in a Dutch school. *Journal of Gender Studies,**33*(4), 375–385. 10.1080/09589236.2023.2242288

[CR51] Krieger, M. A. (2017). Unpacking “sexting”: A systematic review of non-consensual sexting in legal, educational, and psychological literatures. *Trauma, Violence, & Abuse,**18*(5), 593–601. 10.1177/1524838016659486 10.1177/1524838016659486 27436858

[CR52] Lapinski, M. K., & Rimal, R. N. (2005). An explication of social norms. *Communication Theory,**15*(2), 127–147. 10.1111/j.1468-2885.2005.tb00329.x

[CR53] Lippman, J. R., & Campbell, S. W. (2014). Damned if you do, damned if you don’t… if you’re a girl: Relational and normative contexts of adolescent sexting in the United States. *Journal of Children and Media,**8*(4), 371–386. 10.1080/17482798.2014.923009

[CR54] Livingstone, S., Mascheroni, G., Dreier, M., Chaudron, S., & Lagae, K. (2015). *How parents of young children manage digital devices at home: The role of income, education and parental style*. EU Kids Online, London School of Economics and Political Science.

[CR55] Lokot, M., Bhatia, A., Kenny, L., & Cislaghi, B. (2020). Corporal punishment, discipline and social norms: A systematic review in low- and middle-income countries. *Aggression and Violent Behavior,**55*, Article 101507. 10.1016/j.avb.2020.101507

[CR56] Ma, T.-L., Meter, D. J., Chen, W.-T., & Lee, Y. (2019). Defending behavior of peer victimization in school and cyber context during childhood and adolescence: A meta-analytic review of individual and peer-relational characteristics. *Psychological Bulletin,**145*(9), 891–928. 10.1037/bul0000205 31343187 10.1037/bul0000205

[CR57] Mabry, A., & Turner, M. M. (2016). Do sexual assault bystander interventions change men’s intentions? Applying the theory of normative social behavior to predicting bystander outcomes. *Journal of Health Communication,**21*(3), 276–292. 10.1080/10810730.2015.1058437 26716826 10.1080/10810730.2015.1058437

[CR58] Maes, C., Van Ouytsel, J., & Vandenbosch, L. (2024). Active bystanders in the forwarding of sexting messages: Applying a theory of planned behavior in youth. *New Media & Society*, *28*, 312–332. 10.1177/14614448241287729

[CR59] Mainwaring, C., Gabbert, F., & Scott, A. J. (2023a). A systematic review exploring variables related to bystander intervention in sexual violence contexts. *Trauma, Violence, & Abuse,**24*(3), 1727–1742. 10.1177/15248380221079660 10.1177/15248380221079660 PMC10240636 35343337

[CR60] Mainwaring, C., Scott, A. J., & Gabbert, F. (2023b). Behavioral intentions of bystanders to image-based sexual abuse: A preliminary focus group study with a university student sample. *Journal of Child Sexual Abuse,**32*(3), 318–339. 10.1080/10538712.2023.2190734 36921125 10.1080/10538712.2023.2190734

[CR61] Mainwaring, C., Scott, A. J., & Gabbert, F. (2024). Facilitators and barriers of bystander intervention intent in image-based sexual abuse contexts: A focus group study with a university sample. *Journal of Interpersonal Violence,**39*(11–12), 2655–2686. 10.1177/08862605231222452 38281130 10.1177/08862605231222452

[CR62] Marks, L. R., Jenkins, L., Perez-Felkner, L., Templeton, D. P., & Verma, K. (2024). Social cognitive predictors of bystander intervention in racial microaggressions among college students. *Race and Social Problems*, *16*, 249–262. 10.1007/s12552-024-09412-2

[CR63] Martin-Criado, J.-M., Casas, J.-A., Ortega-Ruiz, R., & Del Rey, R. (2021). Supervisión parental y víctimas de ciberbullying: Influencia del uso de redes sociales y la extimidad online. *Revista De Psicodidáctica,**26*(2), 161–168. 10.1016/j.psicod.2020.12.005

[CR64] McMahon, S., & Banyard, V. L. (2012). When can I help? A conceptual framework for the prevention of sexual violence through bystander intervention. *Trauma, Violence, & Abuse,**13*(1), 3–14. 10.1177/1524838011426015 10.1177/1524838011426015 22096017

[CR65] Mesurado, B., Distefano, M. J., Robiolo, G., & Richaud, M. C. (2019). The Hero program: Development and initial validation of an intervention program to promote prosocial behavior in adolescents. *Journal of Social and Personal Relationships,**36*(8), 2566–2584. 10.1177/0265407518793224

[CR66] Mishna, F., Milne, E., Cook, C., Slane, A., & Ringrose, J. (2023). Unsolicited sexts and unwanted requests for sexts: Reflecting on the online sexual harassment of youth. *Youth & Society,**55*(4), 630–651. 10.1177/0044118X211058226

[CR67] Morelli, M., Bianchi, D., & Baiocco, R. (2016). Sexting, psychological distress and dating violence among adolescents and young adults. *Psicothema,**28*(2), 137–142. 10.7334/psicothema2015.193 27112809 10.7334/psicothema2015.193

[CR68] Mori, C., Park, J., Temple, J. R., & Madigan, S. (2022). Are youth sexting rates still on the rise? A meta-analytic update. *Journal of Adolescent Health,**70*(4), 531–539. 10.1016/j.jadohealth.2021.10.026 10.1016/j.jadohealth.2021.10.026 34916123

[CR69] Mosley, M. A., & Lancaster, M. (2019). Affection and abuse: Technology use in adolescent romantic relationships. *American Journal of Family Therapy,**47*(1), 52–66. 10.1080/01926187.2019.1586592

[CR70] Naezer, M., & Van Oosterhout, L. (2021). Only sluts love sexting: Youth, sexual norms and non-consensual sharing of digital sexual images. *Journal of Gender Studies,**30*(1), 79–90. 10.1080/09589236.2020.1799767

[CR71] Neville, F. G. (2014). Preventing violence through changing social norms. In P. D. Donnelly & C. L. Ward (Eds.), *Oxford textbook of violence prevention* (pp. 239–244). Oxford University Press.

[CR72] Ortega, R., Elipe, P., Mora-Merchán, J. A., Calmaestra, J., & Vega, E. (2009). The emotional impact on victims of traditional bullying and cyberbullying: A study of Spanish adolescents. *Zeitschrift für Psychologie / Journal of Psychology,**217*(4), 197–204. 10.1027/0044-3409.217.4.197

[CR73] Panumaporn, J., Hongsanguansri, S., Atsariyasing, W., & Kiatrungrit, K. (2020). Bystanders’ behaviours and associated factors in cyberbullying. *General Psychiatry,**33*(3), Article e100187. 10.1136/gpsych-2019-100187 32524075 10.1136/gpsych-2019-100187 PMC7245368

[CR74] Paquette, M.-M., Dion, J., Bőthe, B., Girouard, A., & Bergeron, S. (2022). Heterosexual, cisgender, and gender- and sexually diverse adolescents’ sexting behaviors: The role of body appreciation. *Journal of Youth and Adolescence,**51*(2), 278–290. 10.1007/s10964-021-01568-z 35098426 10.1007/s10964-021-01568-z

[CR75] Patchin, J. W., & Hinduja, S. (2020). Sextortion among adolescents: Results from a national survey of U.S. youth. *Sexual Abuse,**32*(1), 30–54. 10.1177/1079063218800469 30264657 10.1177/1079063218800469

[CR76] Pistoni, C., Martinez Damia, S., Alfieri, S., Marta, E., Confalonieri, E., & Pozzi, M. (2023). What are the predictors of sexting behavior among adolescents? The positive youth development approach. *Journal of Adolescence,**95*(4), 661–671. 10.1002/jad.12142 36717108 10.1002/jad.12142

[CR77] Polanco-Levicán, K., & Salvo-Garrido, S. (2021). Bystander roles in cyberbullying: A mini-review of who, how many, and why. *Frontiers in Psychology,**12*, Article 676787. 10.3389/fpsyg.2021.676787 34122273 10.3389/fpsyg.2021.676787 PMC8194816

[CR78] Pöyhönen, V., Juvonen, J., & Salmivalli, C. (2010). What does it take to stand up for the victim of bullying? The interplay between personal and social factors. *Merrill-Palmer Quarterly,**56*(2), 143–163. 10.1353/mpq.0.0046

[CR79] Reynolds-Tylus, T., Lukacena, K. M., & Quick, B. L. (2019). An application of the theory of normative social behavior to bystander intervention for sexual assault. *Journal of American College Health,**67*(6), 551–559. 10.1080/07448481.2018.1499648 30285573 10.1080/07448481.2018.1499648

[CR80] Ricon, T., & Dolev-Cohen, M. (2024). Sexting behavior by young adults: The correlation between emotion regulation and moral judgment. *American Journal of Sexuality Education*, *19*, 211–229. 10.1080/15546128.2023.2212189

[CR81] Rimal, R. N. (2008). Modeling the relationship between descriptive norms and behaviors: A test and extension of the theory of normative social behavior (TNSB). *Health Communication,**23*(2), 103–116. 10.1080/10410230801967791 18443998 10.1080/10410230801967791

[CR82] Rimal, R. N., & Lapinski, M. K. (2015). A re-explication of social norms, ten years later: Social norms. *Communication Theory,**25*(4), 393–409. 10.1111/comt.12080

[CR83] Rimal, R. N., Lapinski, M. K., Cook, R. J., & Real, K. (2005). Moving toward a theory of normative influences: How perceived benefits and similarity moderate the impact of descriptive norms on behaviors. *Journal of Health Communication,**10*(5), 433–450. 10.1080/10810730591009880 16199387 10.1080/10810730591009880

[CR84] Rimal, R. N., & Real, K. (2005). How behaviors are influenced by perceived norms: A test of the theory of normative social behavior. *Communication Research,**32*(3), 389–414. 10.1177/0093650205275385

[CR85] Rimal, R. N., & Yilma, H. (2022). Descriptive, injunctive, and collective norms: An expansion of the theory of normative social behavior (TNSB). *Health Communication,**37*(13), 1573–1580. 10.1080/10410236.2021.1902108 33761815 10.1080/10410236.2021.1902108

[CR86] Ringrose, J., Gill, R., Livingstone, S., & Harvey, L. (2012). *A qualitative study of children, young people and “sexting”: A report prepared for the NSPCC*. http://www.nspcc.org.uk/

[CR87] Ringrose, J., Harvey, L., Gill, R., & Livingstone, S. (2013). Teen girls, sexual double standards and ‘sexting’: Gendered value in digital image exchange. *Feminist Theory,**14*(3), 305–323. 10.1177/1464700113499853

[CR88] Rudnicki, K., Vandebosch, H., Voué, P., & Poels, K. (2023). Systematic review of determinants and consequences of bystander interventions in online hate and cyberbullying among adults. *Behaviour & Information Technology,**42*(5), 527–544. 10.1080/0144929X.2022.2027013

[CR89] Salmivalli, C. (2014). Participant roles in bullying: How can peer bystanders be utilized in interventions? *Theory into Practice,**53*(4), 286–292. 10.1080/00405841.2014.947222

[CR90] Salmivalli, C., Lagerspetz, K., Björkqvist, K., Österman, K., & Kaukiainen, A. (1998). Bullying as a group process: Participant roles and their relations to social status within the group. *Aggressive Behavior,**22*(1), 1–15. 10.1002/(SICI)1098-2337(1996)22:1<1::AID-AB1>3.0.CO;2-T

[CR91] Sarmiento, A., Herrera-López, M., & Zych, I. (2019). Is cyberbullying a group process? Online and offline bystanders of cyberbullying act as defenders, reinforcers and outsiders. *Computers in Human Behavior,**99*, 328–334. 10.1016/j.chb.2019.05.037

[CR92] Sasson, H., & Mesch, G. (2016). Gender differences in the factors explaining risky behavior online. *Journal of Youth and Adolescence,**45*(5), 973–985. 10.1007/s10964-016-0465-7 27016219 10.1007/s10964-016-0465-7

[CR93] Schultze-Krumbholz, A., Hess, M., Pfetsch, J., & Scheithauer, H. (2018). Who is involved in cyberbullying? Latent class analysis of cyberbullying roles and their associations with aggression, self-esteem, and empathy. *Cyberpsychology: Journal of Psychosocial Research on Cyberspace*. 10.5817/CP2018-4-2

[CR94] Sedlander, E., & Rimal, R. N. (2019). Beyond individual-level theorizing in social norms research: How collective norms and media access affect adolescents’ use of contraception. *Journal of Adolescent Health,**64*(4), S31–S36. 10.1016/j.jadohealth.2018.12.020 10.1016/j.jadohealth.2018.12.020 PMC6426727 30914165

[CR95] Silva, T. E. D. A., Pereira, R. D. G., & Baltieri, D. A. (2020). Empathy and sexual impulsiveness among medical students who admit to sexting partners’ intimate images. *Journal of Human Growth and Development,**30*(1), 111–119. 10.7322/jhgd.v30.9967

[CR96] Strassberg, D. S., McKinnon, R. K., Sustaíta, M. A., & Rullo, J. (2013). Sexting by high school students: An exploratory and descriptive study. *Archives of Sexual Behavior,**42*(1), 15–21. 10.1007/s10508-012-9969-8 22674035 10.1007/s10508-012-9969-8

[CR97] Suler, J. (2004). The online disinhibition effect. *CyberPsychology & Behavior,**7*(3), 321–326. 10.1089/1094931041291295 15257832 10.1089/1094931041291295

[CR98] Tamarit, A., Schoeps, K., Peris-Hernández, M., & Montoya-Castilla, I. (2021). The impact of adolescent internet addiction on sexual online victimization: The mediating effects of sexting and body self-esteem. *International Journal of Environmental Research and Public Health,**18*(8), 4226. 10.3390/ijerph18084226 33923552 10.3390/ijerph18084226 PMC8072783

[CR99] Thornberg, R., & Jungert, T. (2013). Bystander behavior in bullying situations: Basic moral sensitivity, moral disengagement and defender self-efficacy. *Journal of Adolescence,**36*(3), 475–483. 10.1016/j.adolescence.2013.02.003 23522703 10.1016/j.adolescence.2013.02.003

[CR100] Toomey, R. B., McGuire, J. K., & Russell, S. T. (2012). Heteronormativity, school climates, and perceived safety for gender nonconforming peers. *Journal of Adolescence,**35*(1), 187–196. 10.1016/j.adolescence.2011.03.001 21481925 10.1016/j.adolescence.2011.03.001

[CR101] Van Cleemput, K., Vandebosch, H., & Pabian, S. (2014). Personal characteristics and contextual factors that determine “helping”, “joining in”, and “doing nothing” when witnessing cyberbullying. *Aggressive Behavior,**40*(5), 383–396. 10.1002/ab.21534 24838667 10.1002/ab.21534

[CR102] Van Ouytsel, J., Ponnet, K., Walrave, M., & d’Haenens, L. (2017a). Adolescent sexting from a social learning perspective. *Telematics and Informatics,**34*(1), 287–298. 10.1016/j.tele.2016.05.009

[CR103] Van Ouytsel, J., Van Gool, E., Walrave, M., Ponnet, K., & Peeters, E. (2017b). Sexting: Adolescents’ perceptions of the applications used for, motives for, and consequences of sexting. *Journal of Youth Studies*, *20*, 446–470. 10.1080/13676261.2016.1241865

[CR104] Varava, K. (2019). Children and unhealthy food consumption: An application of the theory of normative social behavior. *Health Communication,**34*(10), 1183–1191. 10.1080/10410236.2018.1471334 29733228 10.1080/10410236.2018.1471334

[CR105] Villacampa, C. (2017). Teen sexting: Prevalence, characteristics and legal treatment. *International Journal of Law, Crime and Justice,**49*, 10–21. 10.1016/j.ijlcj.2017.01.002

[CR106] Wachs, S., Wright, M. F., Gámez-Guadix, M., & Döring, N. (2021). How are consensual, non-consensual, and pressured sexting linked to depression and self-harm? The moderating effects of demographic variables. *International Journal of Environmental Research and Public Health,**18*(5), 2597. 10.3390/ijerph18052597 33807667 10.3390/ijerph18052597 PMC7967514

[CR107] Walker, K., & Sleath, E. (2017). A systematic review of the current knowledge regarding revenge pornography and non-consensual sharing of sexually explicit media. *Aggression and Violent Behavior,**36*, 9–24. 10.1016/j.avb.2017.06.010

[CR108] Walrave, M., Heirman, W., & Hallam, L. (2014). Under pressure to sext? Applying the theory of planned behaviour to adolescent sexting. *Behaviour & Information Technology,**33*(1), 86–98. 10.1080/0144929X.2013.837099

[CR109] Walrave, M., Ponnet, K., Van Ouytsel, J., Van Gool, E., Heirman, W., & Verbeek, A. (2015). Whether or not to engage in sexting: Explaining adolescent sexting behaviour by applying the prototype willingness model. *Telematics and Informatics,**32*(4), 796–808. 10.1016/j.tele.2015.03.008

[CR110] Wilkinson, Y., Whitfield, C., Hannigan, S., Azam Ali, P., & Hayter, M. (2016). A qualitative meta-synthesis of young peoples’ experiences of ‘sexting.’ *British Journal of School Nursing,**11*(4), 183–191. 10.12968/bjsn.2016.11.4.183

[CR111] Wilson, C., Van Steen, T., Akinyode, C., Brodie, Z. P., & Scott, G. G. (2021). To sext or not to sext. The role of social-cognitive processes in the decision to engage in sexting. *Journal of Social and Personal Relationships,**38*(4), 1410–1429. 10.1177/0265407521995884

[CR112] Woodward, V. H., Evans, M., & Brooks, M. (2017). Social and psychological factors of rural youth sexting: An examination of gender-specific models. *Deviant Behavior,**38*(4), 461–476. 10.1080/01639625.2016.1197020

[CR113] Wright, M. F., & Wachs, S. (2024). Longitudinal associations between different types of sexting, adolescent mental health, and sexual risk behaviors: Moderating effects of gender, ethnicity, disability status, and sexual minority status. *Archives of Sexual Behavior*, *53*, 1115–1128. 10.1007/s10508-023-02764-7 38216785 10.1007/s10508-023-02764-7

[CR114] Yamawaki, N. (2007). Rape perception and the function of ambivalent sexism and gender-role traditionality. *Journal of Interpersonal Violence,**22*(4), 406–423. 10.1177/0886260506297210 17369444 10.1177/0886260506297210

[CR115] Yarnell, L. M., Pasch, K. E., Brown, H. S., Perry, C. L., & Komro, K. A. (2014). Cross-gender social normative effects for violence in middle school: Do girls carry a social multiplier effect for at-risk boys? *Journal of Youth and Adolescence,**43*(9), 1465–1485. 10.1007/s10964-014-0104-0 24567165 10.1007/s10964-014-0104-0 PMC4130766

[CR116] Zaikman, Y., & Marks, M. J. (2014). Ambivalent sexism and the sexual double standard. *Sex Roles,**71*(9–10), 333–344. 10.1007/s11199-014-0417-1

[CR117] Zhang, A. T., Land, L. P., & Dick, G. (2010). *Key influences of cyberbullying for university students*. *83*. http://aisel.aisnet.org/pacis2010/83

[CR118] Zhao, Y., Chu, X., & Rong, K. (2023). Cyberbullying experience and bystander behavior in cyberbullying incidents: The serial mediating roles of perceived incident severity and empathy. *Computers in Human Behavior,**138*, Article 107484. 10.1016/j.chb.2022.107484

[CR119] Zhong, L. R., Kebbell, M. R., & Webster, J. L. (2020). An exploratory study of technology-facilitated sexual violence in online romantic interactions: Can the internet’s toxic disinhibition exacerbate sexual aggression? *Computers in Human Behavior,**108*, Article 106314. 10.1016/j.chb.2020.106314

